# Energetics and Kinetics of S-State Transitions Monitored by Delayed Chlorophyll Fluorescence

**DOI:** 10.3389/fpls.2019.00386

**Published:** 2019-03-29

**Authors:** Ivelina Zaharieva, Holger Dau

**Affiliations:** Department of Physics, Freie Universität Berlin, Berlin, Germany

**Keywords:** delayed chlorophyll fluorescence, protonation dynamics, S-state cycle intermediates, thermodynamics, activation energies

## Abstract

Understanding energetic and kinetic parameters of intermediates formed in the course of the reaction cycle (*S*-state cycle) of photosynthetic water oxidation is of high interest and could support the rationale designs of artificial systems for solar fuels. We use time-resolved measurements of the delayed chlorophyll fluorescence to estimate rate constants, activation energies, free energy differences, and to discriminate between the enthalpic and the entropic contributions to the decrease of the Gibbs free energy of the individual transitions. Using a joint-fit simulation approach, kinetic parameters are determined for the reaction intermediates in the *S*-state transitions in buffers with different pH in H_2_O and in D_2_O.

## Introduction

Water oxidation is one of the chemical reactions with largest impact on the planet as it is responsible for the production of the atmospheric O_2_. Recently understanding of the mechanism of water oxidation became of increasing importance, as this reaction is the ultimate source of protons and electrons to be used in to synthesize renewable fuels. Water oxidation takes place in thylakoid membranes of higher plants, algae and cyanobacteria (Ort and Yocum, 1996; Blankenship, 2002; Nelson and Junge, 2015), where it is catalyzed by a manganese-calcium complex embedded in the highly conserved protein environment of Photosystem II protein, PSII (Nelson and Yocum, 2006; Williamson et al., 2011; Pagliano et al., 2013). Breakthroughs in protein crystallography revealed location and structure of the oxygen-evolving complex (OEC), which consists of Mn_4_Ca(μ-O)_5_ cluster surrounded by specific sidechains and functionally important water molecules (Zouni et al., 2001; Ferreira et al., 2004; Umena et al., 2011; Suga et al., 2015; Kern et al., 2018). In spite of recent progress in crystallographic characterization (e.g., Kern et al., 2018) and advanced biophysical investigation of PSII function (e.g., Klauss et al., 2012), the characteristics and mechanistic role of intermediates in the reaction cycle of water oxidation is insufficiently understood. Resolving and understanding transiently formed intermediates of the PSII water oxidation cycle could pave the road toward a complete atomistic picture of the mechanism of photosynthetic water oxidation.

**FIGURE 1:**
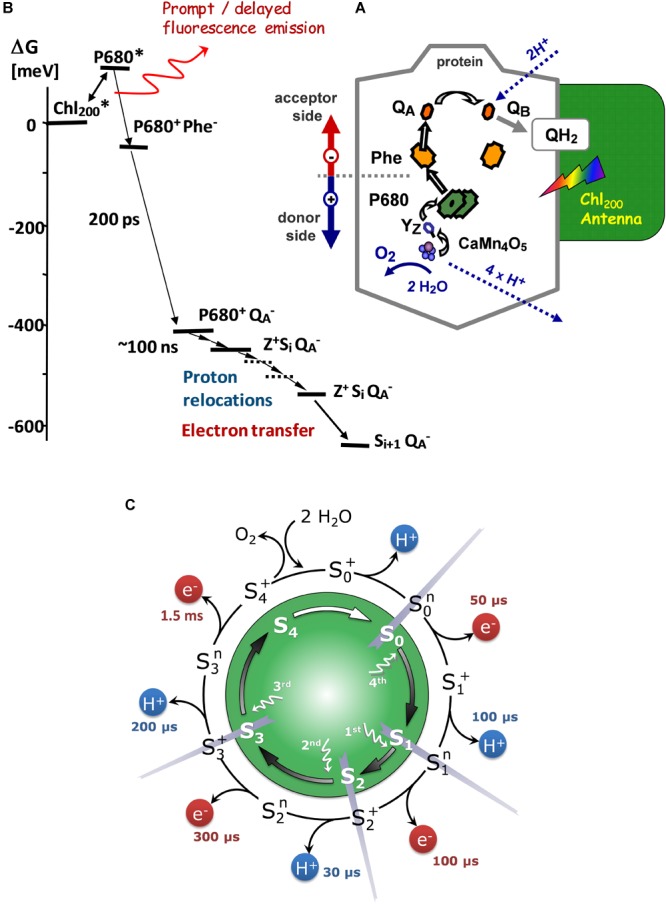
**(A)** Scheme of the sequence of light-induced reactions in PSII **(B)** Energy-level diagram of radical-pair states in PSII. The zero-level of the Gibbs free-energy scale is chosen to correspond to the excited state of the chlorophyll antenna, which is lower than the P680^∗^ level due to the antenna entropy contribution (Dau, 1994; Dau and Sauer, 1996). Modified from Buchta et al. (2007). **(C)** S-state cycle of the photosynthetic water oxidation. Inner circle: The classical Kok model (Kok et al., 1970). Outer circle: Extended *S*-cycle describing both oxidation of the Mn complex by electron transfer to the Y_Z_^•+^ radical and proton removal from the Mn complex or its ligand environment (Based on Klauss et al., 2012).

When exposing dark-adapted photosynthetic organisms (or isolated photosystems) to a sequence of saturating flashes of visible light, a characteristic patter of period-of-four oscillations is observed for the flash-number dependence of O_2_-formation (Joliot et al., 1969). This characteristic flash pattern has been explained by Kok et al. (1970) by the basic *S*-state cycle model describing the accumulation of four “positive charges” before onset of dioxygen formation and assuming 5 intermediate states labeled *S_0_* to *S_4_* (Kok et al., 1970). Later it has been realized that not only electrons are removed from the Mn_4_CaO_5_ cluster (by the Tyr161 of the D1 protein, Tyr161, Y_Z_, Faller et al., 2001, 2003) but also protons are removed from the OEC (Junge et al., 2002) so that the extent of charge accumulation depends on the temporal sequence of electron and proton removal steps. Consequently, Kok’s basic reaction cycle does not describe the accumulation of four of four positive charges, but of four oxidizing equivalents before the onset of the O-O bond formation. In 2007 an extended S-state cycle was proposed that explicitly takes into account electron and proton removal steps, where alternating electron-proton removal prevents charge accumulation and a prohibitive increase in the redox potential of the active-site metal complex, as illustrated in Figure 1 (Dau and Haumann, 2007a,b). Clear experimental evidences for *S*-state cycle intermediates in the extended S-state cycle is available only for some of the reactions (Haumann et al., 2005; Gerencser and Dau, 2010; Klauss et al., 2012; Zaharieva et al., 2016). For all transiently formed intermediates, their energetic, kinetic and structural properties are insufficiently understood. Addressing the knowledge gaps regarding the reaction kinetics, motivates our study.

Fluorescence measurements are highly informative method to address the function of Photosystem II, as they can provide a variety of information about the photosynthetic apparatus in its native state (Kalaji et al., 2014, 2017). Typically, the variability of the chlorophyll (Chl) fluorescence yield is recorded after sudden application of an actinic light, in the time range form microseconds to tens of minutes (fluorescence induction curves). However, often high complexity hinders quantitative interpretation in terms of reaction kinetics of specific molecular events seriously. This is even more the case for the delayed chlorophyll fluorescence (*DF*, recombination fluorescence) (Goltsev et al., 2009). Thus, we use laser-flash excitation for investigation of the events in PSII. Unlike induction kinetics, the decays measured after nanosecond laser-flash illumination of dark-adapted samples provide better-defined starting conditions and a more direct access to information about the reactions taking place in PSII at various times after flash-excitation.

Light excitation of dark adapted PSII results in the formation of excited singlet states of Chl followed by rapid excitation energy transfer and equilibration among the antenna chlorophylls of PSII (rapid exciton equilibration) (Schatz et al., 1988; Dau and Sauer, 1996). Excitation of the chloropyll(s) denoted as P680 results in formation of P680^∗^, which initiates the primary charge separation and leads to reduction of a specific pheophytin (Phe) and formation of P_680_^+^, the oxidized primary donor (Figure 1). The pheophytin is reoxidized by electron transfer to the primary quinone acceptor, Q_A_. The P_680_^+^ states are “quenched” mostly by a donation of an electron from the primary electron donor, Y_Z_, to P_680_^+^ within less than 1 μs after the Laser flash. The Y_Z_ oxidation is followed by an oxidation of the Mn_4_CaO_5_ cluster coupled by structural rearrangements and proton-removal steps. Q_A_^-^ decays are mostly due to electron transfer to Q_B_ at the acceptor side, a clearly slower process with a half-time of several milliseconds.

The chlorophyll fluorescence intensity is proportional to the fraction of excited antennae-chlorophyll molecules [Chl^∗^] and to the probability to decay via fluorescence emission. For the chlorophyll fluorescence (prompt fluorescence, PF), which is measured as a fluorescence intensity during illumination, there is a clear relation between the probability for charge separation (formation of P680^+^Q_A_^-^ radical pair) and the PF intensity, manifested in increase of PF with accumulation of reduced Q_A_^-^ and vise versa (Dau, 1994). When measurements are performed in darkness, and the time interval between the light excitation and detection is in the microsecond or millisecond domain (and thus clearly longer than the lifetime of excited chlorophyll molecules), PF is not detectable but a weak fluorescence emission (delayed fluorescence, *DF*) that relates to repopulation of the Chl^∗^ state by recombination of charge-separated radical pairs can be recorded. The actual population of the Chl^∗^-state in this case is determined by the free-energy difference between the excited-antenna state and the radical-pair state [Yz^+^Q_A_^-^] reached at the respective time (Buchta et al., 2007; Goltsev et al., 2009):

$$\begin{array}{c} DF(t)\sim [chl^{*}]=[TyrY_{z}^{•+}Q_{A}^{-}](t)e^{\Delta G_{A•RP}}/k_{B}T\;Eq.\;1.1 \end{array}$$

Where ΔG_A^∗^RP_ is the difference in the Gibbs free energy (Δ*G* < 0) between the excited antenna (Chl^∗^) and the PSII radical-pair state [Yz^•+^Q_A_^-^], k_B_ is the Boltzmann constant, and T is the absolute temperature in Kelvin.

The rapid decrease in the *DF* fluorescence measured in the dark after a flash-excitation, is due to the decrease in the concentration of [Yz^•+^Q_A_^-^] states by (i) electron transfer from Q_A_ to Q_B_ (reoxidation of the reduced acceptor (Q_A_^-^), (ii) reduction of Y_Z_^•+^ by electron transfer from the Mn_4_CaO_5_ complex and (iii) charge recombination between Y_Z_^•+^ and Q_A_^-^, where the latter is a competing process that depends on the concentrations of the [Yz^•+^Q_A_^-^] state and thus also on the first two reactions (Goltsev et al., 2009). Also, completion of electron transfer to Y_Z_^•+^ does not eliminate *DF* emission completely, because a fraction of PSII will still harbor Y_Z_, according to the Gibbs free energy difference of this electron transfer step (Figure 1). This means that after the time required for an electron transfer from Mn_4_CaO_5_ cluster to Y_Z_^•+^, there is a fraction of reaction centers where Y_Z_^•+^ is still present. Moreover, *DF*(t) decreases because proton movements or structural changes stabilize the radical pair. The way all these processes affect the *DF* signal can be generalized: *DF*(t) reflects the total Gibbs free energy differences (toward the excited antenna state) of any state formed in the reaction sequence. If we can identify the decrease in the *DF* decay to a transition between state-A and state-B in the *DF* decay (e.g., by assignment of an exponential decay), the corresponding ΔG_AB_ can be calculated from the *DF* level of state-A, *DF*(A), and the (lower) *DF* level of state-B, *DF*(B), according to:

$$\begin{array}{c} \Delta G_{AB}=-k_{B}T\ln\frac{DF(A)}{DF(B)}\;Eq.\;1.2 \end{array}$$

Here *DF*(A) corresponds to the *DF* level before onset of the A→B transition and *DF*(B) corresponds the *DF* level after its completion. Using this rationale, analysis of the *DF* signal can provide number not only for the time constants of the transition from state-A to state-B, but also for the corresponding ΔG_AB._

In Buchta et al. (2007) a rationale was developed to correct *DF* decays measured after ns-Laser flash excitation for the contribution of Q_A_^-^ reoxidation by combined measurements of *DF* decays and prompt fluorescence decay under similar experimental conditions. Applying this method, the *DF* decay can be used to obtain information mostly about the processes at the donor side of PSII, related to the proton and electron transfer at the Mn_4_CaO_5_ cluster. The bottleneck in using the potentially highly informative *DF* analysis is the complexity of the *DF* decays, where the individual kinetic components contributing to the total *DF* decay are difficult to resolve.

In the present work, we analyze decays of the *DF* fluorescence of PSII extending from 10 μs to 10 ms. Using a multi-exponential model, a high-quality fit typically requires minimally four exponential components, resulting in nine independent fit parameters. Typically, parameter correlations will render several of these nine parameters uncertain. Therefore, in past studies, mostly a three-exponential model was employed (Haumann et al., 2005; Buchta et al., 2007; Zaharieva et al., 2011) for simulation of the third-flash decays. Then the resulting fit parameters were used to calculate three potentially informative figures: the time-constant of O_2_ formation, the total Gibbs free energy of the proton removal steps, and a mean time-constant for all proton removal steps. These three figures are largely insensitive to fit-parameter correlations, but especially the inability to resolve the time-constants of the proton removal step individually represents a serious drawback. In our study, a joint-fit simulation approach that allows to overcome, at least partially, this limitation is presented.

## Materials and Methods

### Sample Preparation

Highly active PSII membrane particles were prepared from market spinach according to Schiller and Dau (2000). The rate of O_2_ evolution under continuous illumination with white light at 28°C was about 1000–1400 μmol.mg chl^-1^.h^-1^ and the Chl *a*/Chl *b* ratio was about 2.5. After preparation the membrane particles were stored at -80°C in buffer A: 25 mM MES (2- morpholinoethanesulfonic acid, 99.5%, Merck, pK_a_ = 6.15), 10 mM NaCl (≥99.8%, Roth), 5 mM MgCl_2_ (≥99%, Roth), 5 mM CaCl_2_ (≥98%, Roth), 1 M glycine-betaine (betaine-monohydrate, Alfa Aesar, 99%), pH 6.4 (adjusted with NaOH). The PSII partic1es were stored in Ependorff vials at a chlorophyll concentration of 2–3 mg/ml.

Before measurements, 1 ml PSII suspension (chlorophyll concentration of about 2 mg chl.ml^-1^) was thawed on ice (for 60 min, in the dark) and gently resuspended in 2–3 ml buffer A using a soft brush. The volume was adjusted to 40 ml with Buffer A and the protein sample centrifuged 12 min at 48 000 *g* in order to remove residuals of starch and free chlorophyll. The pellet was resuspended in 3–4 milliliters of buffer B (25 mM MES, 25 mM MOPS (3-morpholinopropanesulfonic acid, 99,5%, AppliChem, pK_a_ = 4.0), 25 mM HEPPS (4-(2-hydroxyethyl) piperazine-1-propanesulfonic acid, 98%, AppliChem, pK_a_ = 8.0), 10 mM NaCl, 5 mM MgCl_2_, 5 mM CaCl_2_, 1 M glycine-betaine, pH adjusted to 6.4 or 5.2 with concentrated solutions of NaCl or HCl). To account the different sensitivity of the standard pH electrode to protons and deuterons, a correction to the value read from the pH meter in D_2_O buffer (pH’) was done according to pD = pH’ +0.4 (Glasoe and Long, 1960).

Chlorophyll concentration was determined in 80% acetone-water mixture according to Lichtenthaler (1987) using Cary 50 Conc UV-Vis Spectrophotometer, Varian. The chlorophyll concentration was adjusted with Buffer B to 100 μg/ml and the diluted suspension stored in ice in dark during the *DF* measurements, but no longer that 1 h. One minute before the measurements directly in a cuvette the PSII suspension was mixed with Buffer B and DCBQ [2,6-dichloro-1,4-benzoquinone, Aldrich, 98%, 1% DCBQ in water-DMSO mixture (dimethylsulfoxid, Merck)] to a final concentration of 10 μg chl/ml, 20 μmol DCBQ and final volume of 1.5 ml.

### Time-Resolved Measurements of Prompt Chl Fluorescence

The yield of the prompt fluorescence for excitation with a sequence of laser-Continuum Minilite II (532 nm, FWHM of 5 ns, 2 mJ/cm^2^, 700 ms interval between flashes) was detected at various times in a pump-probe experiment by means of commercially available instrument (FL 3000, Photon Systems Instruments) and used for correction of the *DF* decays in order to remove the contribution of the Q_A_^-^ decay due to Q_A_^-^ reoxidation by Q_B_ (acceptor side contribution) as described elsewhere (Buchta et al., 2007).

### Time-Resolved Delayed Fluorescence Measurements

Delayed fluorescence measurements were performed by an in-house setup as described in Grabolle and Dau (2005). For the measurements, a polystyrene (PS) cuvette (BRAND, 340–900 nm, length path 10 mm) with four clear sides was placed in cuvette holder made from copper and facilitating temperature control by a flow-through system. By means of a water bath thermostat (Circulator DC50, bath K40, HAAKE), the temperature of the PSII suspension was set at a fixed value in the range between 0 and 30°C, with an estimated accuracy of 0.2°C. The dark-adapted samples were excited by a saturating Laser flash of 2 mJ/cm^2^ (Continuum Minilite II, 532 nm, FWHM of 5 ns, time between flashes of 0.7 s). The delayed fluorescence signal was recorded by a gated photomultiplier (Hamamatsu R2066; PMT Gated Socket Assembly C1392-55; anode voltage, 1000 V; anode resistor, 2.2 kOhm; gating voltage, 240 V applied from 7 μs before to 3 μs after the Laser flash). Scattered Laser light was suppressed by a combination of two long-pass filters (LINOS Photonics, DT-red and DT-magenta with cut-off wavelength 600 and 632 nm, respectively). After amplification (Tektronix AM502, bandwidth of 300 kHz), the signal was sampled at 1 MHz by a 12-bit PC-card (ADLINK, PCI 9812). At least three repetitions were done for each measurement and averaged after applying the corrections described below. The reproducibility of the data is illustrated in the Supplementary Figure S10.

### Data Analysis

The *DF* decays obtained after each Laser flash were corrected for an artifact of the detector system as described elsewhere (Grabolle and Dau, 2005). The artifact predominantly was due to excitation of delayed fluorescence in the glass or cathode material of the photomultiplier, by the strong prompt fluorescence of the PSII particles as well as by scattered light of the excitation flash. Additionally, the *DF* signal was corrected for the contribution from the [Q_A_^-^] decay (Buchta et al., 2007).

The obtained *DF* decay curve was simulated as a sum of four exponential decays plus an offset (Eq. 2.1).

$$\begin{array}{c} F\left(t\right)=\sum_{i=1}^{4}a_{i}\exp\left(-t/\tau_{i}\right)+c\;Eq.\;2.1 \end{array}$$

The parameters *a_i_, τ_i_*, and *c* (9 parameters in total for each *DF* decay) were determined by minimization of the error sum. For curve-fitting and in all figures, we averaged the delayed fluorescence decays “logarithmically” such that a linear spacing of the points on a logarithmic time axis was achieved. Then the error sum was calculated according to:

$$\begin{array}{c} \varepsilon^{2}=\sum_{N}{\left(\log\frac{F_{sim}}{F_{sim}}\right)}^{2}\;Eq.\;2.2 \end{array}$$

In order to avoid overparametrization, a joint simulation approach was developed as detailed in the “Results” section. Simulations were done by in-house software developed by Dr. P. Chernev (Chernev, 2007) using Object Pascal programming language and Simulated Annealing minimization algorithm (van Laarhoven and Aarts, 1987).

## Results

### Simulation Approach

**FIGURE 2:**
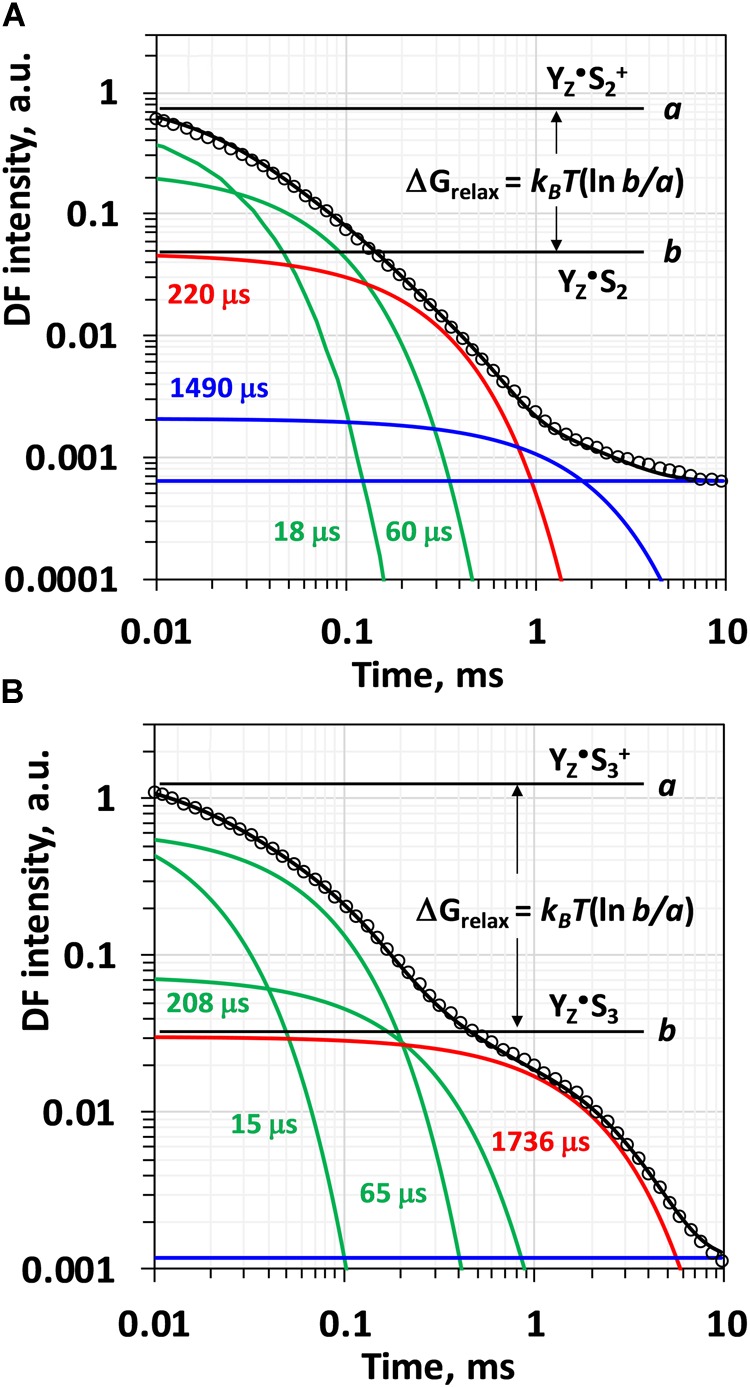
Simulation of the delayed fluorescence decay measured after second **(A)** and after third **(B)** ns-flash excitation of dark adapted PSII particles at 20°C and pH 6.4. Experimental points are shown with circles, simulated curves according to Eq. 2.1 within the joint fit simulation approach as solid black lines. The individual components are also shown: green lines and corresponding time constants represent the components used to simulate the multiphasic process of intermediate formation associated with H^+^ release step, red lines represent the electron transfer step (Mn_4_CaO_5_ → Y_Z_^•+^) and blue lines model the slow reactions presumably not related to processes at the donor side of PSII. The change in the Gibbs free energy, Δ*G*, is calculated according the equations 3.3–3.6 from the ratio of the levels before and after the reaction of interest (the case shown in the plot is for the total free energy change, Δ*G_relax_*, for the reactions that take place before the electron transfer step).

Visual inspection of the *DF* decays recorded after the third flash reveals a biphasic behavior (Figure 2). The first phase extends over the first 0.3 ms and has been associated with intermediate formation by proton removal from the oxygen evolving complex by a deprotonation step during the *S_3_ → S_0_* transition (Haumann et al., 2005; Buchta et al., 2007). The second step with a halftime of about 1.7 ms is assignable to the electron transfer step associated with dioxygen formation (Klauss et al., 2012). Simulation with a sum of two exponential decays only, however, has been proven to be clearly unsatisfactory (Buchta et al., 2007). Excellent agreement between experimental data and simulated curve can be achieved only by four-exponential fit (Supplementary Figure S1) suggesting that the deprotonation step is not a simple single-phase process, but at least three, presumably sequential reaction steps are involved (Buchta et al., 2007). Using four exponentials according to Eq. 2.1 would require 9 free parameters (5 amplitudes and 4 lifetimes) to simulate a single *DF* decay and the fit will be strongly undetermined. This would hamper the determination of the individual rate constants as well as the calculation of the free-energy change associated with each individual process. That is why so far only the total (summed) amplitude of the first decays was used to calculate the total free energy change associated with the *S_4_* intermediate formation (as shown in Figure 2), exploiting the relative independence of the sum of the individual amplitudes (pre-exponential factors) on the simulation approach (Buchta et al., 2007; Zaharieva et al., 2013).

To obtain in a more reliable way the time constants of all intermediate steps, a joint-fit approach for simulation of set of *DF* decays measured at different temperatures was proposed in Zaharieva et al. (2013): *DF* decays measured at 7 different temperatures (same data set used also in the present study) were simulated implementing the Arrhenius equation to interrelate the rate constants at different temperatures:

$$\begin{array}{c} k_{i}=1/\tau_{i}=k_{0_{i}}\exp\left(-E_{a_{i}}/k_{B}T\right)\;Eq.\;3.1 \end{array}$$

In this way instead of four different rate constants for each of the 7 different temperatures, only 4 pre-exponential (frequency) factors (*k*_0*i*_) and 4 activation energies (*E_ai_*) were used. These 8 parameters are the same for all temperatures, assuming linear Arrhenius plots in the temperature range from 0 to 30°C. The linear Arrhenius plot is predicted by standard electron-transfer as well as transition-state theory and largely has been experimentally confirmed for the reactions in the oxygen-evolving complex of PSII (Klauss et al., 2012; Bao and Burnap, 2015; Klauss et al., 2015; Gates et al., 2016). Using this rationale, instead of 7 × 9 = 63 adjustable parameter for the set of 7 *DF* decays measured at different temperatures, the overall number of parameters was reduced to 8 + 5 × 7 = 43 free parameters (Zaharieva et al., 2013).

Although this approach allowed for more reliable determination of the rate constants, the precise determination of the amplitudes and thus the change in the free energy associated to the individual intermediates was not possible. Instead, only the total change in Gibbs free energy was determined using the sum of the individual amplitudes, as shown in Figure 2. In this study, we approach the thermodynamic parameters of the individual transitions implementing another restrain to the joint-fit simulation:

$$\begin{array}{c} \Delta G_{i}=\Delta H_{i}-T\Delta S_{i}\;Eq.\;3.2 \end{array}$$

Using an analogy to Eq. 1.2 and the approach presented in Figure 2, the change of the Gibbs free energy for the individual reaction steps can be expressed as:

$$\begin{array}{c} \Delta G_{4}=-k_{B}T\ln\frac{a_{4}+c}{c}\;Eq.\;3.3\\ \Delta G_{3}=-k_{B}T\ln\frac{a_{3}+a_{4}+c}{a_{4}+c}\;Eq.\;3.4\\ \Delta G_{2}=-k_{B}T\ln\frac{a_{2}+a_{3}+a_{4}+c}{a_{3}+a_{4}+c}\;Eq.\;3.5\\ \Delta G_{1}=-k_{B}T\ln\frac{a_{1}+a_{2}+a_{3}+a_{4}+c}{a_{2}+a_{3}+a_{4}+c}\;Eq.\;3.6 \end{array}$$

where a_i_ denote the amplitudes of the individual kinetic components and *c* is constant (see Eq. 2.1). This allows us to calculate the amplitude of each component, a_i_, from ΔG_i_ and the amplitudes of the rest of the kinetic components. Eq. 3.2 allows us to calculate the individual amplitudes as function of the changes in enthalpy and entropy during the reaction using the set of Eqs. 3.3–3.6. In this way the constant from Eq. 2.1 remains the only parameter to be determined by free variation during the simulation. In addition, changes in the enthalpy, ΔH, and entropy, ΔS, associated to each transition are explicitly added to the model (but they are independent on the temperature). For the entire temperature set of 7 decays considered in the joint-fit approach this results in replacement of 5 × 7 = 35 amplitudes by 7 constants and 2 thermodynamics parameters for each of the 4 kinetic components, or 15 independent parameters. The total number of free parameters for the set of 7 *DF* decays measured at 7 different temperatures thus decreases to 23 [4 frequency factors, 4 activation energies, 4 enthalpies, 4 entropic contributions and 7 constants (offsets)].

Using the simulation approach outlined here, the average number of free parameters used for each single decay decreases from 9 in the free 4 exponentials fit to 23/7 = 3.28, or it approaches the number of the free parameters for the case of single exponential decay. A comparison of the residuals from the fit using different number of exponentials and the joint-fit model is shown in the Supplementary Figure S1. We note that the measurements at different temperatures are independent, and fresh PSII sample was used for each of them.

### DF Decays After the Third Flash (*S_*3*_ → S_*0*_ +* O_2_ Transition)

The *DF* decays analyzed in this work were detected by applying a sequence of saturating ns-Laser flashes to dark adapted PSII membrane particles and published in the Ph.D. thesis of Markus Grabolle (Grabolle, 2005). After the third Laser flash, the majority of PSII centers undergo *S_3_ → S_0_* transition (Dau and Haumann, 2008). Delayed fluorescence decays measured after the third flash were simulated according to Eq. 2.1, where the slowest component represents the electron transfer step and O-O bond formation (Figure 1, 2). This slow electron transfer step is well resolved and easily identified already by visual inspection as a plateau level in the *DF* decay reached about 300 μs after the flash (Figure 2B). It is now well established, that this process of Mn oxidation is preceded by proton removal from the OEC (Haumann et al., 2005; Dau and Haumann, 2007b; Klauss et al., 2012; Siegbahn, 2013). Previously it has been suggested that there could be three sequential reaction steps relating to proton removal form the OEC that occur before the onset of Mn reduction in the dioxygen-formation step (Dau and Haumann, 2007b). In order to model adequately the processes of proton and electron transfer occurring after the third flash in PSII, four exponentials are needed. The first three of them are related to the fast process of proton removal, but they are mathematically difficult to resolve. The very similar values obtained for the rate constants of these reactions when simulating the *DF* decay measured at 20°C independently (k_i_^-1^ equals to 17, 72, and 284 μs) and within the joint fit approach (k_i_^-1^ equals to 15, 65, and 208 μs, Supplementary Figure S1) confirm the validity of the model applied for the joint-fit analysis. Previously lifetimes of 14, 65, and 203 μs in free four-exponential fit of *DF* decays measured under similar conditions were reported (Buchta et al., 2007).

**FIGURE 3:**
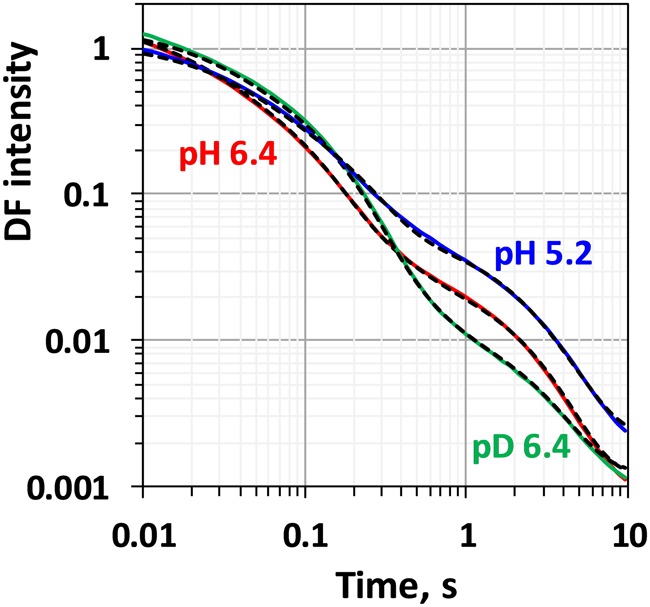
Delayed fluorescence decays measured after the third flash at 20°C in H_2_O buffer with pH 6.4 (red line), pH 5.2 (blue line) and in D_2_O buffer with pD 6.4 (green line). Black dashed lines represent the simulated curves according to Eq. 2.1 within the joint fit simulation approach. Simulation parameters are shown in Table 1, Figure 4, 5 and Supplementary Figure S3.

**FIGURE 4:**
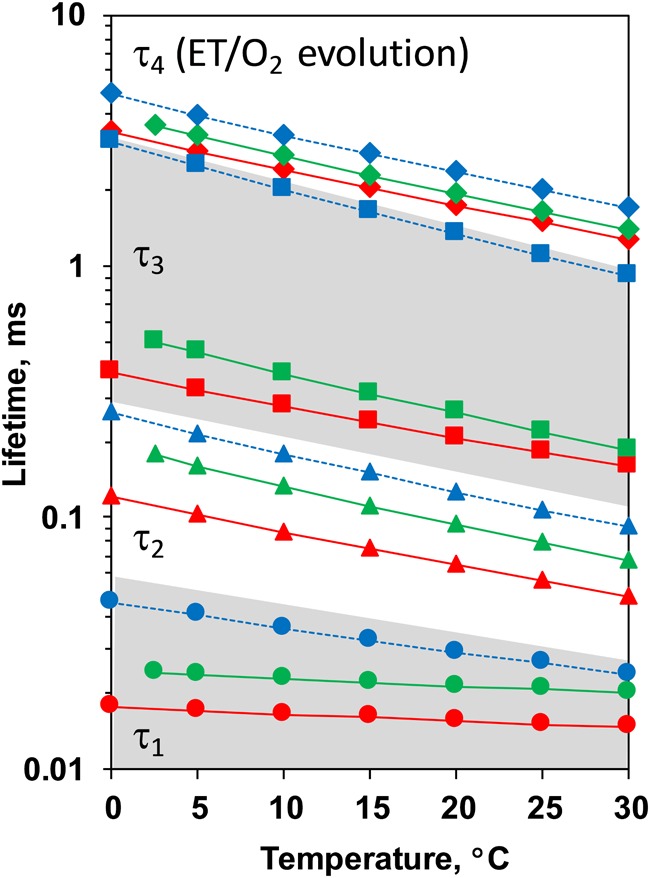
Time constants of the *DF* decays measured after the third ns-Laser flash excitation of dark adapted PSII particles at pH 6.4 (red), pD 6.4 (green) and pH 5.2 (blue). The areas of the first and third kinetic components are shadowed in gray.

Comparison between the decays measured at 20°C at pH 6.4 and 5.2 as well as in H_2_O and D_2_O is shown in Figure 3. (Full set of *DF* decays measured at all temperatures, including the simulated curves is shown in the Supplementary Figure S2; The confidence intervals of the fit parameters, as calculated from the covariance matrix of system, are shown in Supplementary Table S2). Time constants of the individual kinetic components (Figure 4) show that both D_2_O and a decrease of pH result in slowing down of all reactions. This effect is most drastic in the third kinetic component where the decrease of pH results in a change of τ_3_ from 208 μs at pH 6.4 to 1.33 ms at pH 5.2 (values for 20°C, see Table 1). The least affected from isotope exchange or pH drop is the electron transfer step (the slowest reaction).

**Table 1:** Time constants (τ), activation energies (*E_*a*_*) and further thermodynamic parameters: Gibbs free energy (Δ*G*), enthalpy (Δ*H*) and the entropic contribution to the change in Gibbs free energy (*T*Δ*S*), as determined from simulations of the *DF* after the third ns-Laser flash applied to dark-adapted PSII membrane particles.

S-state transition	S3 → S0	ET/PT	τ (μs)	Ea (meV)	ΔG (meV)	ΔH (meV)	-TΔS (meV)
H_2_O, pH 6.4	S3^+^ → S3^n^	H^+^	15	46	–19	–82	63
			65	217	–49	156	–205
			208	204	–30	–56	26
	S3^n^ → S0^+^	e^-^ (O_2_)	1736	234	–83	31	–114
D_2_O, pD 6.4	S3^+^ → S3^n^	H^+^	21	47	–16	–91	74
			93	253	–59	189	–248
			260	258	–40	–107	67
	S3^n^ → S0^+^	e^-^ (O_2_)	1934	248	–64	39	–103
H_2_O, pH 5.2	S3^+^ → S3^n^	H^+^	29	158	–21	2	–23
			127	249	–53	44	–97
			1330	294	–19	–22	2
	S3^n^ → S0^+^	e^-^ (O_2_)	2358	245	–64	–32	–32

*For the time constants, *ΔG*, and *T*Δ*S*, the values for 20°C are presented.*

The slope of the temperature dependence of the time constants remains almost unaffected for all components expect for the fastest one at pH 5.2 (Figure 4). This indicates that there is no significant change in the activation energies, *E_*a*_*, of the reactions. The total activation energy of the proton removal step, *E_a_*_relax_, was 156 meV at pH 6.4 and in qualitative agreement with the previously determined value of 180 meV (Barry et al., 2006; Zaharieva et al., 2013). *E_a_*_relax_ at pD 6.4 was similar (184 meV) and increased to a value of 277 meV at pH 5.2 (Table 1). The activation energy of 234 meV obtained for the dioxygen formation step (Table 1) agrees well with the previously determined value of 231 meV, using *DF* data (Buchta et al., 2007) as well as with the value of 230 meV, determined using photothermal beam deflection (PDB) data (Klauss et al., 2012) and time resolved absorption changes at 355 nm (Renger and Hanssum, 1992). However, for still unclear reasons, the *E_*a*_* determined here is significantly lower than previously determined activation energies of 340 meV (near-UV data in Clausen et al., 2004), 420 meV (polarographic data Clausen et al., 2004), and 380 meV (dye-sensitized absorption measurements Haumann and Junge, 1994).

Kinetic isotope effect (KIE) for the H/D exchange was extensively studied before as KIE values higher than unity helps to identify the processes that involve proton movements. Values of 2.4 for the KIE for the H^+^ removal step were obtained from optical absorption spectroscopy (Gerencser and Dau, 2010) and 2.5 from time-resolved X-ray spectroscopy (Zaharieva et al., 2016). For the O-O bond formation step lower values of 1.2 were determined by optical spectroscopy (Gerencser and Dau, 2010), 1.3 by photothermal beam deflection (Klauss et al., 2012) and about 1.4 by X-ray absorption spectroscopy (Zaharieva et al., 2016). Here, we obtain lower values of 1.6 for the H^+^ removal step and 1.1 for the electron transfer step. We note that in order to facilitate the comparison to previous studies, instead of calculating the ratio between the rate constants of the individual kinetic components, we calculated the KIE of the H^+^ release as a ratio between the mean lifetimes for all reactions before the oxygen formation step [τ_mean_ = (τ_1_.*a*_1_ + τ_2_. *a*_2_ + τ_3_. *a*_3_)/(*a*_1_+ *a*_2_ *a*_3_)]. Although it is not clear if these systematically lower values are due to the specifics of the *DF* measurements or are imposed by the analysis, the trend of observing higher KIE for the proton release step is confirmed.

**FIGURE 5:**
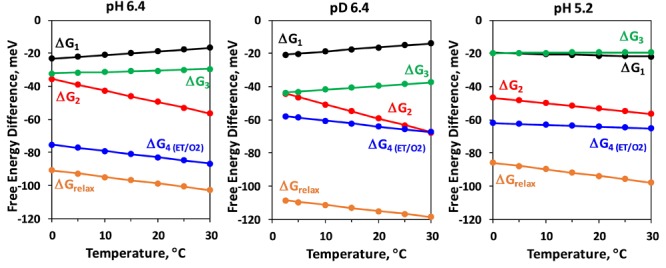
Temperature dependence of the Gibbs energy, Δ*G_i_*, of the individual kinetic components resolved in the *DF* decay after the third Laser flash applied to dark adapted PSII particles. Δ*G_relax_*, assignable to formation of a reaction intermediate after Y_Z_ oxidation but before the electron transfer from Mn_4_CaO_5_ complex and O_2_ evolution is also shown (for calculation of Δ*G_relax_* see Figure 2).

The temperature dependence of the Gibbs free energy, Δ*G*, is shown in Figure 5. Notably, both the isotope exchange and pH decrease have a strong effect on Δ*G* of the electron transfer step (Δ*G_4_*), significantly decreasing the change of Δ*G* as compared to the more natural control conditions (pH 6.4). Pronounced effect of D_2_O exchange and pH decrease on the free energy change of the reactions associated with H^+^ release, Δ*G_relax_*, was reported earlier (Haumann et al., 2005), but on contrast to the electron transfer step, with D_2_O increasing the free energy drop, and low pH decreasing the change in Δ*G_relax_*. These results were reproduced in this study. Our results further show that these effects are mostly due to changes in the third kinetic component with lifetime of about 200 μs (Figure 5).

The negative slope of the temperature dependence of Δ*G* is related to the entropy increase during the reaction. This trend is observed for both, the H^+^ release step (Δ*G_relax_*) and for the electron transfer step (Δ*G_4_*) for all conditions. Figure 5 shows that while Δ*G* is always negative, some of the kinetic steps are characterized with positive slope of the temperature dependence and thus are associated with decrease of entropy. This is the case for first (15 μs) and third (200 μs) kinetic components at pL 6.4. Decrease of pH results in strong decrease of the entropic factor (Figure 5). The thermodynamic parameters obtained from the simulation are summarized in Table 1.

### DF Decays After the Second Flash (*S_*2*_ → S_*3*_* Transition)

**FIGURE 6:**
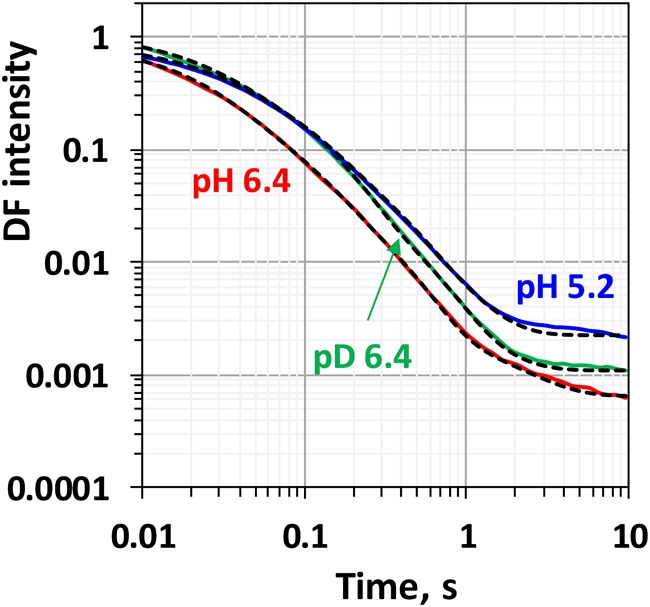
Delayed fluorescence decays measured after the second flash at 20°C in H_2_O buffer with pH 6.4 (red line), pH 5.2 (blue line) and in D_2_O buffer with pD 6.4 (green line). Black dashed lines represent the simulated curves according to Eq. 2.1 within the joint fit simulation approach. Simulation parameters are shown in Table 2, Figure 7, 8 and Supplementary Figure S5.

The second ns-Laser flash excitation applied to dark adapted PSII particles induces the *S_2_ → S_3_* transition at the donor side of PSII (Dau and Haumann, 2008). *DF* decays recorded at 20°C after the second flash are shown in Figure 6 (DF decays measured at all temperatures, as well as for the simulated curves see Supplementary Figure S4; The confidence intervals of the fit parameters are shown in Supplementary Table S3). The *DF* decays were simulated with sum of 4 exponential decays within the joint-fit approach as it was done for the *DF* decays recorded after the 3rd flash (Figure 2A). During the *S_2_ → S_3_* transition proton is released form the Mn_4_CaO_5_ cluster, followed by electron transfer from the Mn_4_CaO_5_ complex to Y_Z_^•+^ (Dau et al., 2012). These processes were modeled by three exponents, assuming that the deprotonation is a bi-phasic process. The last component has very low amplitude (Figure 2A) and is likely related to processes in the acceptor side of the PSII, thus it is not of interest for this study. Although very good fit quality was achieved, in this case mixing of different components is more likely due to the absence of well resolved slow decay component. This affects the significance of the thermodynamic parameters determined by this approach as outlined in the following.

**FIGURE 7:**
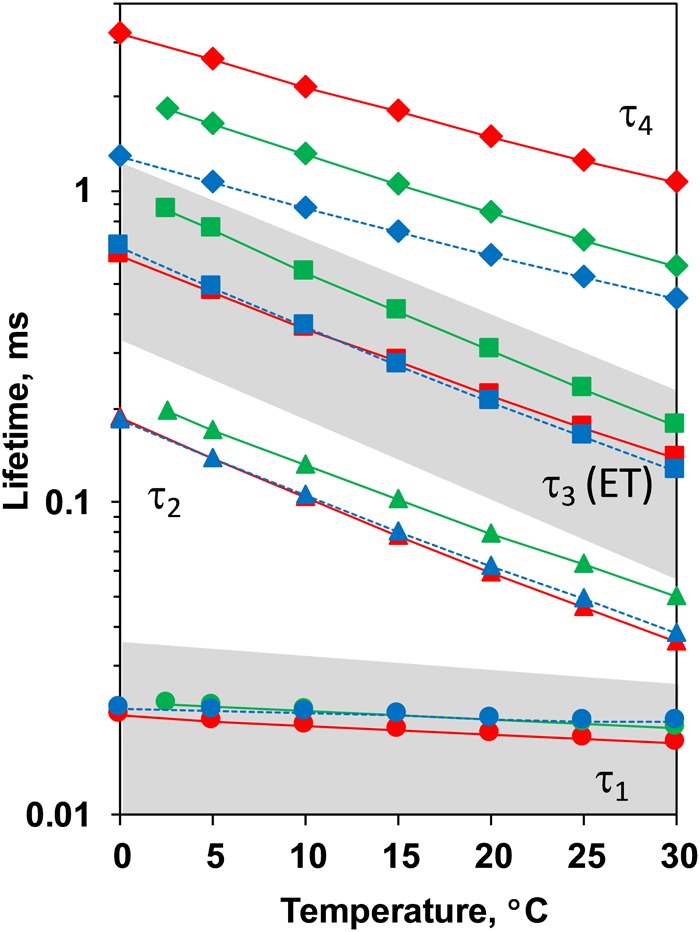
Time constants of the *DF* decays measured after the second ns-Laser flash excitation of dark adapted PSII particles at pH 6.4 (red), pD 6.4 (green) and pH 5.2 (blue). The areas of the first and third kinetic components are shadowed in gray.

The time constants and activation energies determined here agree well with the literature values. The averaged life time of the deprotonation step preceding the electron transfer step, τ*_mean_* = (τ_1_. *a*_1_ + τ_2_. *a*_2_)/(*a*_1_ + *a*_2_) at 20°C equals 29 μs while the value determined by X-ray absorption spectroscopy was 26 μs (Zaharieva et al., 2016) and the value in Klauss et al. (2012) determined by photothermal beam deflection was 30 μs. The activation energy of the reaction determined before using photothermal beam deflection was 470 meV (Klauss et al., 2012) while the activation energies of the two kinetic components related to the deprotonation step determined in this study are 49 and 397 meV (*E_arelax_* = 271 meV) (Table 2). For the electron transfer step we obtained time constant of 220 μs with *E_a_* = 355 meV, in a relatively good agreement with the values reported previously of about 300 μs (Renger and Hanssum, 1992; Haumann et al., 1997; Haumann et al., 2005; Gerencser and Dau, 2010; Zaharieva et al., 2016) with *E_a_* = 360 meV (Renger and Hanssum, 1992; Haumann et al., 1997).

**Table 2:** Time constants (τ), activation energies (*E_a_*) and further thermodynamic parameters: Gibbs free energy (Δ*G*), enthalpy (Δ*H*) and the entropic contribution to the change in Gibbs free energy (*T*Δ*S*), as determined by simulations of the *DF* after the second ns-Laser flash applied to dark-adapted PSII membrane particles.

S-state transition	S2 → S3	ET/PT	τ (μs)	Ea (meV)	ΔG (meV)	ΔH (meV)	–TΔS (meV)
H_2_O, pH 6.4	S2^+^ → S2^n^	H^+^	18	49	–30	10	–40
			60	393	–44	–102	59
	S2^n^ → S3^+^	e^-^	220	355	–74	–121	47
			1490	262	–36	–81	45
D_2_O, pD 6.4	S2^+^ → S2^n^	H^+^	20	47	–22	–15	–7
			79	356	–57	–20	–37
	S2^n^ → S3^+^	e^-^	304	417	–57	–103	46
			850	305	–38	–219	181
H_2_O, pH 5.2	S2^+^ → S2^n^	H^+^	20	23	–15	–32	17
			62	374	–37	–122	85
	S2^n^ → S3^+^	e^-^	209	393	–48	–62	15
			618	249	–52	–137	84

*For the time constants, Δ*G*, and *T*Δ*S*, the values for 20°C are presented.*

Despite the relatively good agreement between the rate constants and activation energies obtained here and reported in literature, there is a disagreement between our data and literature values for the kinetic isotope effect, KIE, calculated as ratio between the rate constants in H_2_O and D_2_O buffers. With KIE for the proton release step of 1.5 (calculated as ratio between τ*_mean_*), we could not reproduce the large KIE of 4.5 (Zaharieva et al., 2016) or 5.6 reported earlier (Klauss et al., 2012). We also obtained KIE of 1.4 for the electron transfer step, a value smaller than the KIE of 1.7 reported before (Gerencser and Dau, 2010; Klauss et al., 2012) or 1.8 (Zaharieva et al., 2016). Still, in our calculations, although moderate in amplitude, the KIE is larger for the deprotonation step than for the electron-transfer step.

Another disagreement between the data presented in Table 2 and literature is related to the especially high entropic contribution for the deprotonation step (large temperature dependence of Δ*G_relax_*) reported earlier based on less restrictive joint-fit analysis of the same dataset (Zaharieva et al., 2013). These findings are at least partially explainable with the difficulties to simulate the rather featureless *DF* decays measured after the second laser flash. Repeating the simulation with different starting values, we were able to get larger KIE of 1.6 for the proton release step and 2.2 for the electron transfer step and to reproduce the steep temperature dependence of ΔG_relax_ with only slight decrease of the fit quality (see Supplementary Figures 6–9 and Supplementary Table 1). In this simulation however, as well as in the simulations in Zaharieva et al. (2013), there is disagreement with the literature values for the rate constants and activation energies as discussed above. These results suggests that even using highly restrictive simulation approach, there is still a large ambiguity in the results from simulation of the *DF* decays after the second flash and the thermodynamic parameters of the S_2_ →S_3_ transition determined by this approach should be discussed with care.

## Discussion

The extended S-state cycle (Figure 1) extends Kok’s classical reaction cycle by considering explicitly the removal of protons from the Mn-complex (Dau and Haumann, 2007a,b). The proton removal step that precedes the electron transfer in the *S_3_ → S_0_* transition was concluded from time-resolved XAS experiments in 2005, when a 200 μs lag-phase (delay) before onset of the Mn reduction that is coupled to O_2_ formation was observed (Haumann et al., 2005). In conjunction with *DF* data reported in the same study and further results (Rappaport et al., 1994; Haumann et al., 2008; Gerencser and Dau, 2010; Rappaport et al., 2011; Klauss et al., 2012, 2015), the time-resolved XAS experiment provided conclusive evidence for alternating proton and electron removal from the Mn_4_CaO_5_ cluster before O-O bond formation, with proton removal preceding the electron transfer step. Experimental evidences for the temporal sequence of events during the *S_2_ → S_3_* transition as proposed by the extended S-cycle model were obtained in 2012 by tracking of the proton-removal step in time-resolved photothermal beam deflection experiments (Klauss et al., 2012, 2015) and in recent time-resolved XAS experiments (Zaharieva et al., 2016). Here, we obtained similar rate constants for both, proton removal and electron transfer steps as determined before. Good agreement was found also with respect to the activation energies of the proton and electron transfer steps, as determined from the temperature dependence of the rate constants (see “Results” section).

Delayed fluorescence decays after saturating ns-Laser-flash excitation allow to go a step further and to resolve additional kinetic components that are currently not accessible by other experimental methods. To do that, a more comprehensive method for analysis, going beyond the simple three-exponential fit is needed and such approach is presented in the present study. Moreover, *DF* decays allow for direct estimation of the ratio between equilibrium populations of the states (equivalent to equilibrium constant) and thus to determine directly the differences in the Gibbs free energy, Δ*G*, as detailed by Eqs. 1.1 and 1.2 and as illustrated by Figure 1B, 2. The rationale has been described in detail also in Grabolle and Dau (2005) and Buchta et al. (2007). The temperature dependence of Δ*G* provides access to the entropic and enthalpic contribution to the total change in Gibbs free energy change, according to the equation 3.2. We note that the values for energetic parameters are not directly related to the activation entropy and activation enthalpy as they can be obtained with the Eyring equation from the temperature dependence of experimentally determined rate constants.

A deprotonation process is predicted to be associated with a sizable or even dominating entropic contribution to the free energy change (*T*Δ*S* > Δ*H*, entropically driven reaction) and positive Δ*H* (endergonic process), due to the entropy increase associated with the “dilution” of the proton released in the bulk water. This is observed for the total change in Gibbs free energy during the Y_Z_^•^*S_3_^+^* → Y_Z_^•^*S_3_^n^* transition (Δ*G_relax_* in Figure 5). As expected, lowering pH (increase of proton concentration) decreases the absolute value of free energy drop associated with the deprotonation, while the H/D isotope exchange has an opposite effect. These results for Δ*G_relax_* during *S_3_* →*S_4_* transition were reported previously (Haumann et al., 2005), but our approach allows for individual analysis of the three kinetic components associated with the deprotonation step. The negative slope in the temperature dependence of Δ*G_relax_* is mirrored by the 65 μs component (Δ*G*_2_), but surprisingly Δ*G_relax_* of the second kinetic component shows almost no dependence on pH or H/D isotope exchange (Figure 5). The pH and H/D dependence is reproduced by the third kinetic component with 200 μs lifetime (Δ*G*_3_, Figure 5). A possible interpretation of these results is that during the *S_3_* →*S_0_* transition the proton release into the aqueous bulk occurs with 65 μs, preceded by (15 μs) rearrangement of a protein-internal H-bonded protein-water network and followed by (200 μs) protein-internal proton relocation from the catalytic core of the OEC toward a nearby site. The slow process is delayed with decrease of pH where the higher degree of protonation hampers the proton relocation within the protein-water matrix (increase of τ_3_ from 200 μs at pH 6.4 to 1.3 ms at pH 5.2, see Table 1). The proton relocation could include proton movements from (substrate) water species bound at the Mn_4_CaO_5_ core of the OEC toward amino acid groups close to Mn_4_CaO_5_ complex. It also may involve proton relocation along proton channels extending toward carboxylate clusters at the protein surface (Haumann and Junge, 1994; Dau and Haumann, 2008; Bondar and Dau, 2012; Karge et al., 2014). The relatively small activation energy, the enthalpy decrease and the small dependence on the pH and isotope exchange of the fastest 15 μs kinetic component implies that it may be related to fast reorganizations e.g., proton movements within hydrogen bonds induced through bond or through space (electric fields) interactions with the photooxidized Y_Z_^•+^ (Styring et al., 2012; Nakamura et al., 2014). The sequence of events thus could be the following:

(1) Light-induced formation of Y_Z_^•+^ is followed by an initial rearrangement of the H-bonded protein-water cluster surrounding the catalytic metal cluster (15 μs).
(2) Long-range electrostatic interactions of the positively charged Y_Z_^•+^ protonated peripheral groups (most likely carboxylate sidechains) result in deprotonation and proton release into the aqueous bulk, possibly (or even likely) followed immediately by proton transfer from the “outskirts” of the OEC toward the previously deprotonated group at the periphery of the PSII protein complex.
(3) A proton is relocated from the core of the metal cluster (Mn_4_CaO_5_ core plus first-sphere water ligands) toward the OEC outskirts (200 μs). Completion of this deprotonation step represents the pre-requisite of subsequent electron transfer and O-O bond formation.
(4) An electron is transferred from the Mn ions of the OEC (Mn oxidation) or directly from a “substrate oxygen” (ligand oxidation) followed immediately by O-O bond formation associated with Mn reduction and eventually O_2_ release (1.7 ms).

**FIGURE 8:**
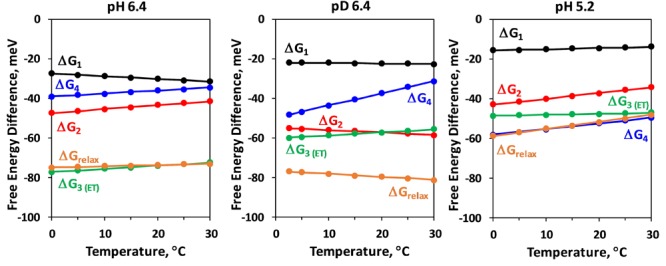
Temperature dependence of the Gibbs energy, Δ*G_i_*, of the individual kinetic components resolved in the *DF* decay after the second ns-Laser flash applied to dark adapted PSII particles. Δ*G_relax_*, assignable to formation of a reaction intermediate after Y_Z_ oxidation but before the electron transfer from Mn_4_CaO_5_ complex is also shown (for calculation of Δ*G_relax_* see Figure 2).

The interpretation of the results for the second laser flash (*S_2_* →*S_3_*) is less straightforward, also because of the ambiguity of the simulation results. In this case we use only two kinetic components to simulate the deprotonation step which precedes the electron transfer from Mn_4_CaO_5_ cluster to Y_Z_^•+^. A clear similarity is observed between the first kinetic component resolved in the *DF* decay after the second and the third flash, in terms of time constant (about 15 μs), surprisingly low activation energy, relative independence on pH and isotope exchange as well as low entropic contribution to the free energy drop (Table 2 and Figure 8). These similarities suggest that in both *S_2_* →*S_3_* transition and *S_3_* →*S_0_* transition, the deprotonation step is initiated by similar rearrangements in the proton bonding network possibly induced by the nearby Y_Z_^•+^. The likely deprotonation step in the Y_Z_^•^*S_2_^+^* → Y_Z_^•^*S_2_^n^* transition (second kinetic component) has similar time constant of about 65 μs as the one found in Y_Z_^•^*S_3_^+^* → Y_Z_^•^*S_3_^n^* transition, but is characterized by a larger activation energy; and it is not an entropically driven reaction (according to Figure 8 and Table 2 there is even a slight decrease in entropy observed in both pH 6.4 and pH 5.2). By the latter characteristics as well as by the strong pH dependence this component is rather similar to the third component resolved in *S_3_* →*S_0_* transition (tentatively assigned to reorganization of H-bonding network and decrease of total energy during Y_Z_^•^*S_3_^+^* → Y_Z_^•^*S_3_^n^* the transition). For the electron transfer in the *S_2_* →*S_3_* we detect values of 200–300 μs, which is slightly faster, but still reasonably close to the figures determined by other methods (Renger and Hanssum, 1992; Haumann et al., 1997; Haumann et al., 2005; Gerencser and Dau, 2010; Zaharieva et al., 2016). Similarly, also the activation energy agrees reasonably well with previously published figures (Renger and Hanssum, 1992; Haumann et al., 1997). However, in clear contrast to other methods that detect the rate constant of electron transfer more specifically, we do not observe any slowing down at lower pH. Moreover, a fourth exponential component of unclear origin is required for high-quality simulations, with time constants at 20°C of 1.5 ms (pH 6.4, H_2_O), 850 μs (pD 6.4, D_2_O) and 620 μs (pH 5.5, H_2_O). We note that the latter value is close to the time constant determined for the electron transfer step in the *S_2_* →*S_3_* transition (at 20°C and pH 5.5) by UV-vis spectroscopy (Gerencser and Dau, 2010). This fourth component might relate to acceptor side processes, but some aspects like the acceleration in D_2_O cannot be explained easily, neither by a donor side nor an acceptor side process. Taking into account these discrepancies, we conclude that most likely the used simulation approach failed to describe the events in the *S_2_* →*S_3_* transition appropriately.

## Data Availability

All datasets generated for this study are included in the manuscript and/or the Supplementary Files.

## Author Contributions

HD initiated the study. IZ and HD designed the model and wrote the manuscript. IZ performed the simulations.

## Conflict of Interest Statement

The authors declare that the research was conducted in the absence of any commercial or financial relationships that could be construed as a potential conflict of interest.
