# Supplementary material for: Energetics and Kinetics of S-State Transitions Monitored by Delayed Chlorophyll Fluorescence
Source: Front Plant Sci. 2019 Mar 29;10:386. doi: 10.3389/fpls.2019.00386 (PMC6450259; doi:10.3389/fpls.2019.00386)
Supplement: Supplementary file 1 [file Data_Sheet_1.PDF]

Supplementary Material

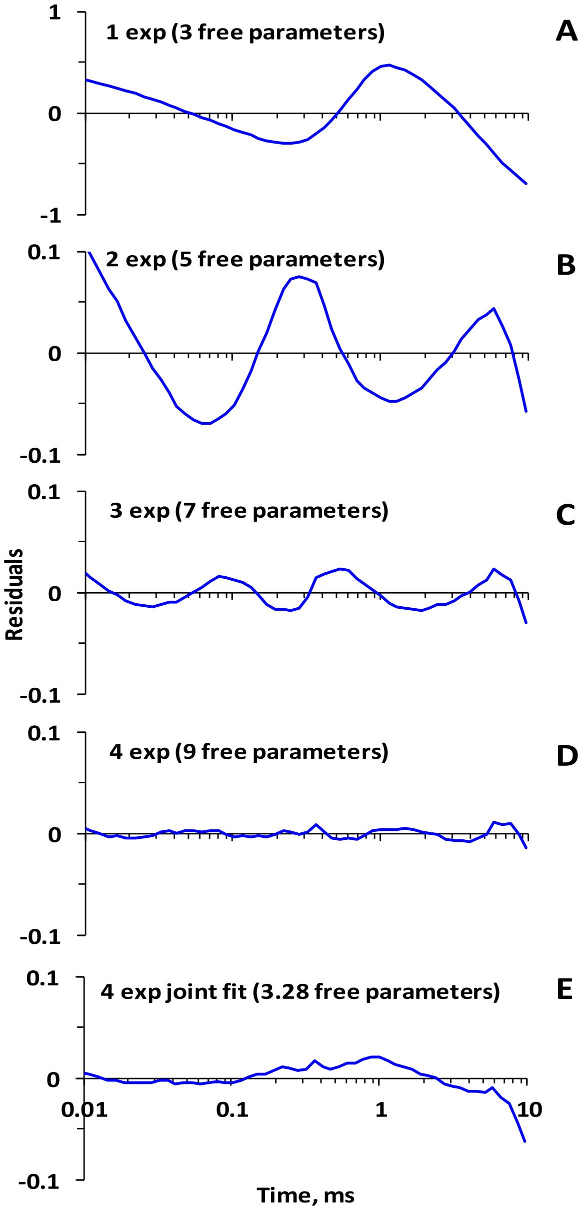

Supplementary Figure S1. Residual plots for simulation of the *DF* decay measured after the third flash at 20 °C and pH 6.4. (A) Simulation as a single exponent with τ = 168 µs. (B) Sum of two exponentials with lifetimes 64 µs and 1.46 ms. (C) Sum of three exponentials with lifetimes 25 µs, 105 µs and 1.62 ms. (D) Sum of four exponents with lifetimes 17 µs, 72 µs, 284 µs and 1.75 ms. (E) Sum of four exponents within the joint fit approach. Lifetimes 15 µs, 65 µs, 208 µs and 1.74 ms. Note the 10-fold larger Y-scale in panel A.

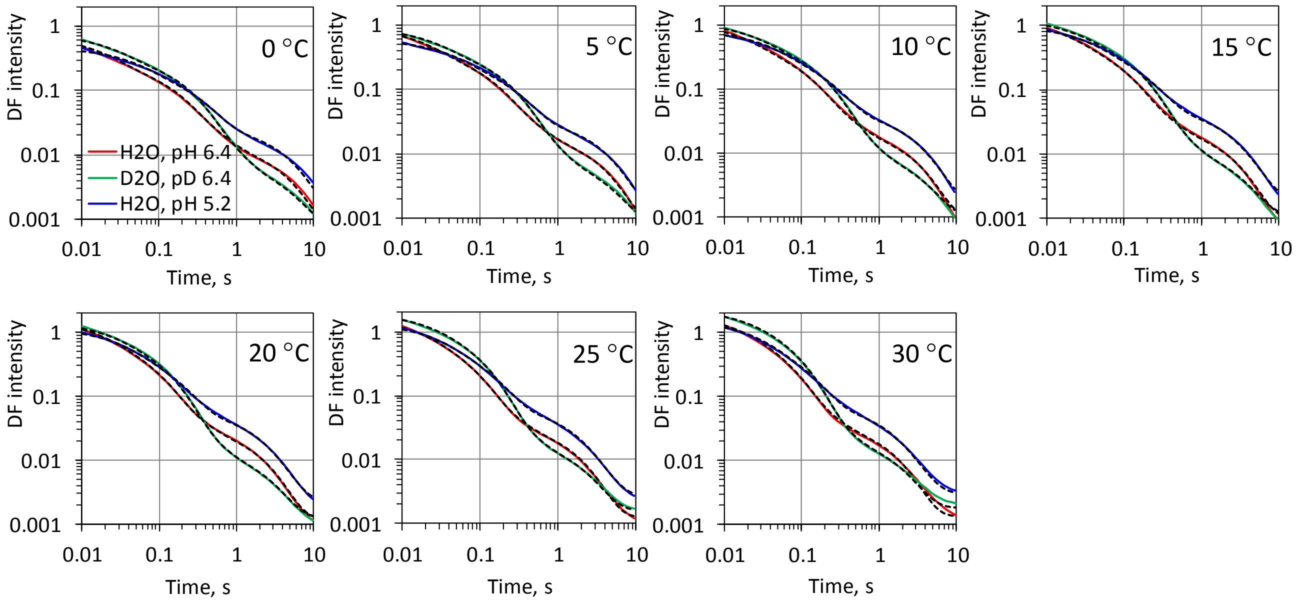

Supplementary Figure S2. Delayed fluorescence decays measured after the third ns-Laser flash excitation of dark adapted PSII samples at different temperatures in H_2O buffer with pH 6.4 (red line), pH 5.2 (blue line) and in D_2O buffer with pD 6.4 (green line). Black dashed lines represent the simulated curves according to Eq. 2.1 within the joint fit simulation approach. Simulation parameters are shown in Figures 4, 5, Table 1 and Supplementary Figure S3.

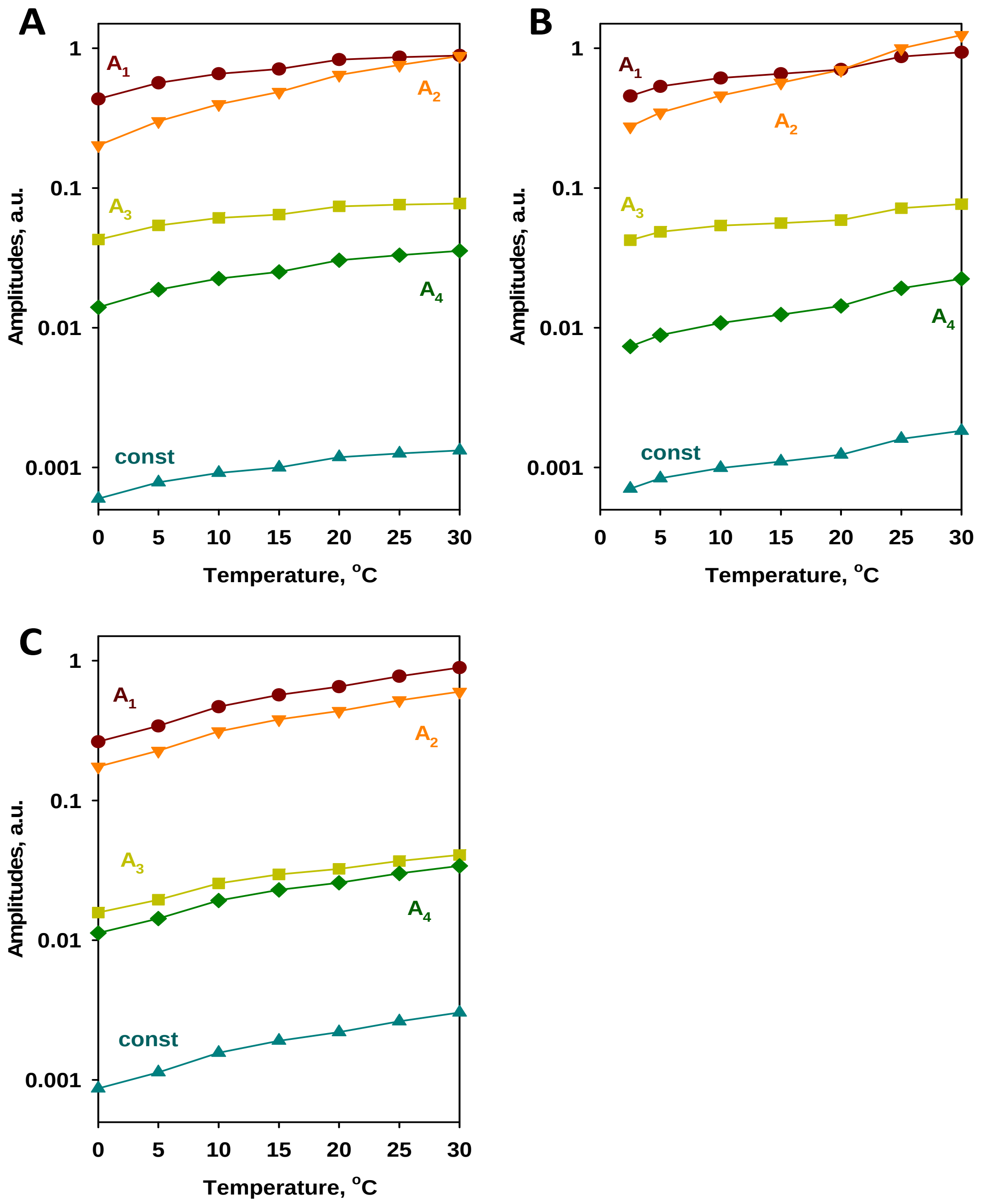

Supplementary Figure S3. Amplitudes of the *DF* decays measured after the third Laser flash excitation of dark adapted PSII particles. (A) in H_2O buffer at pH 6.4. (B) in D_2O buffer at pD 6.4. (C) in H_2O buffer at pH 5.2.

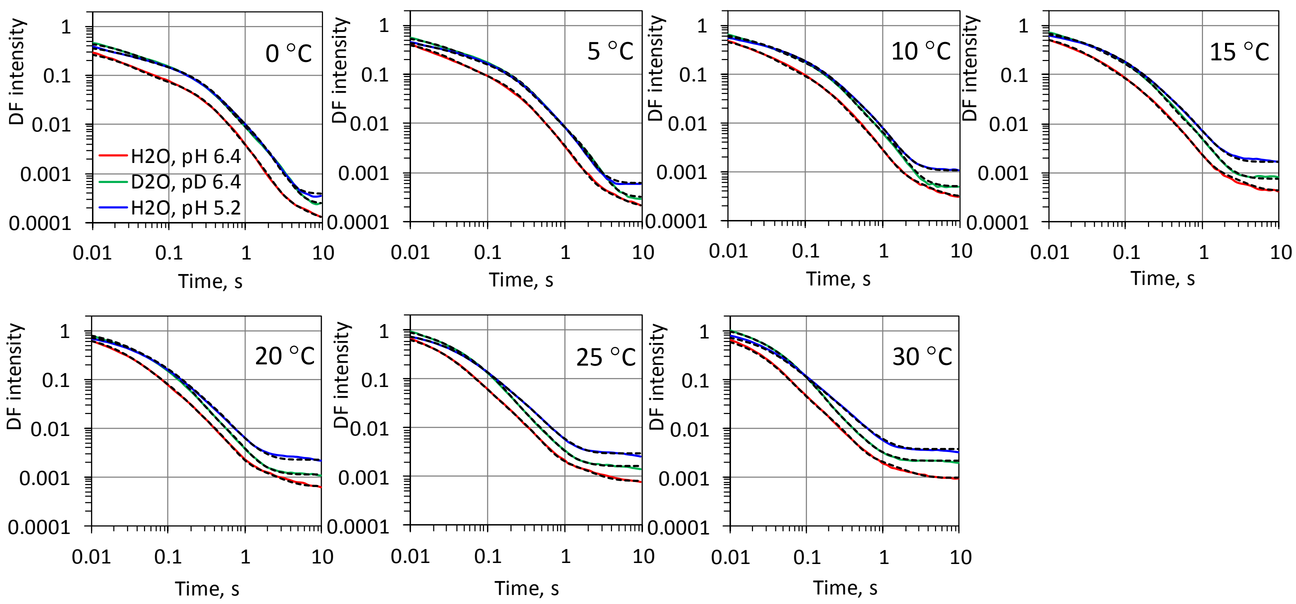

Supplementary Figure S4. Delayed fluorescence decays measured after the second ns-Laser flash excitation of dark adapted PSII samples at different temperatures in H_2O buffer with pH 6.4 (red line), pH 5.2 (blue line) and in D_2O buffer with pD 6.4 (green line). Black dashed lines represent the simulated curves according to Eq. 2.1 within the joint fit simulation approach. Simulation parameters are shown in Figures 7, 8, Table 2 and Supplementary figure S5.

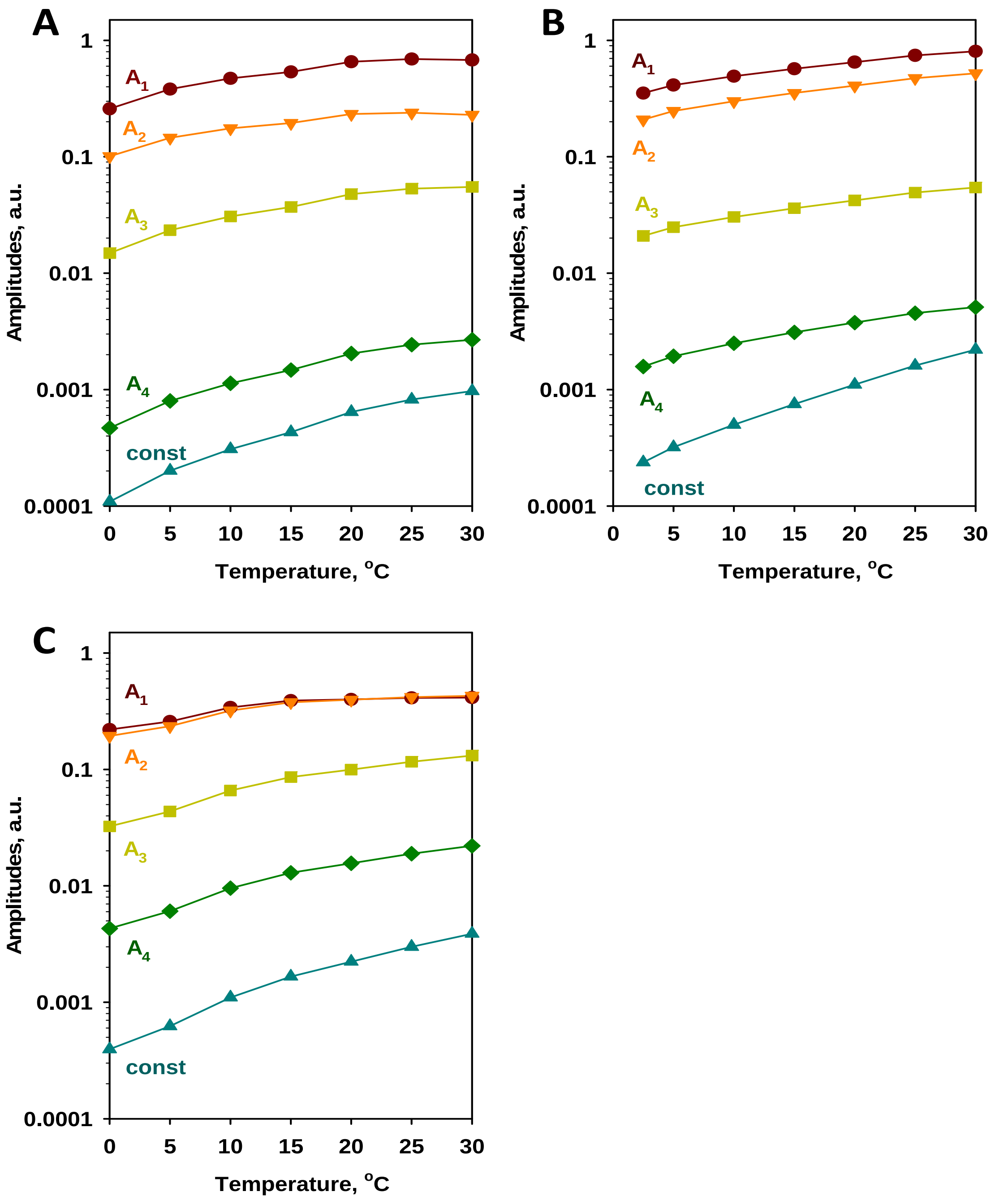

Supplementary Figure S5. Amplitudes of the *DF* decays measured after the second ns-Laser flash excitation of dark adapted PSII particles. (A) in H_2O buffer at pH 6.4. (B) in D_2O buffer at pD 6.4. (C) in H_2O buffer at pH 5.2.

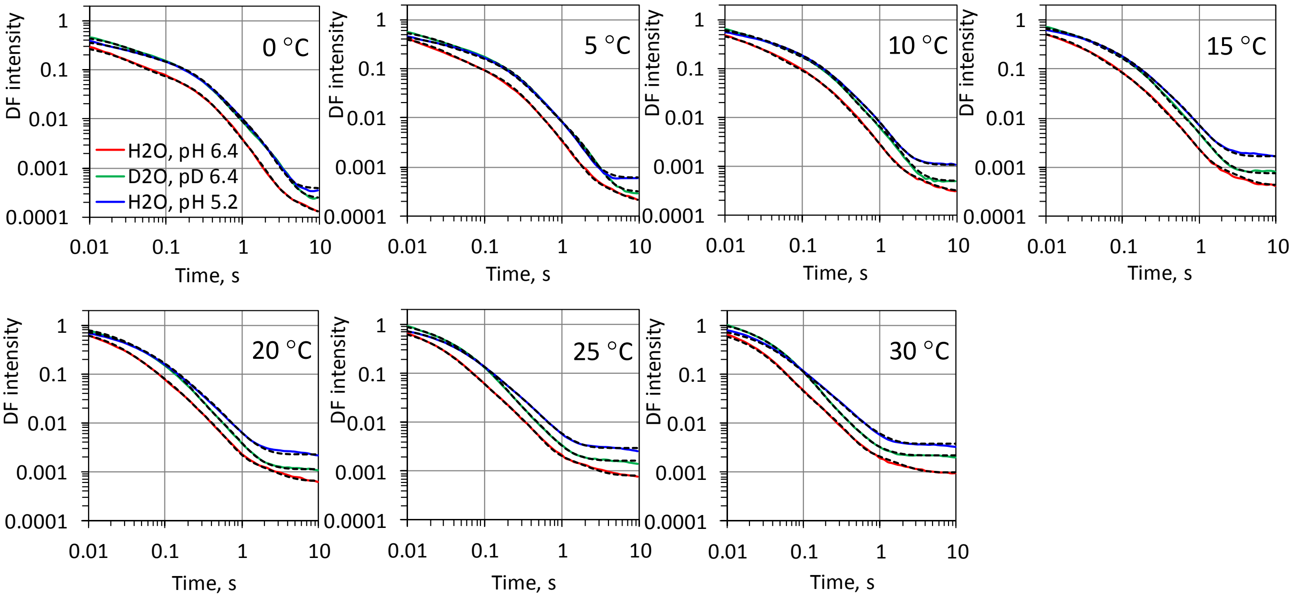

Supplementary Figure S6. Delayed fluorescence decays measured after the second ns-Laser flash excitation of dark adapted PSII samples at different temperatures in H_2O buffer with pH 6.4 (red line), pH 5.2 (blue line) and in D_2O buffer with pD 6.4 (green line). Black dashed lines represent the simulated curves according to Eq. 2.1 within the joint fit simulation approach. Simulation parameters are shown in Supplementary Figures S7, S8, S9 and Supplementary Table 1.

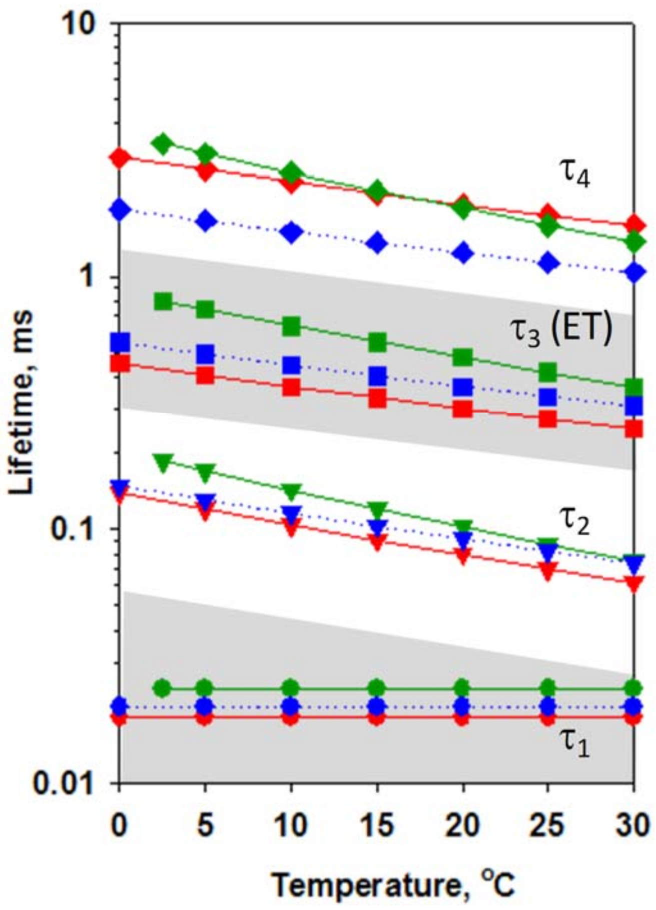

Supplementary Figure S7. Time constants of the *DF* decays measured after the second ns-Laser flash excitation of dark adapted PSII particles at pH 6.4 (red), pD 6.4 (green) and pH 5.2 (blue). The areas of the first and third kinetic components are shadowed in grey. These simulation parameters are related to the second simulation, presented in Supplementary Figures S6, S8, S9 and Supplementary Table 1.

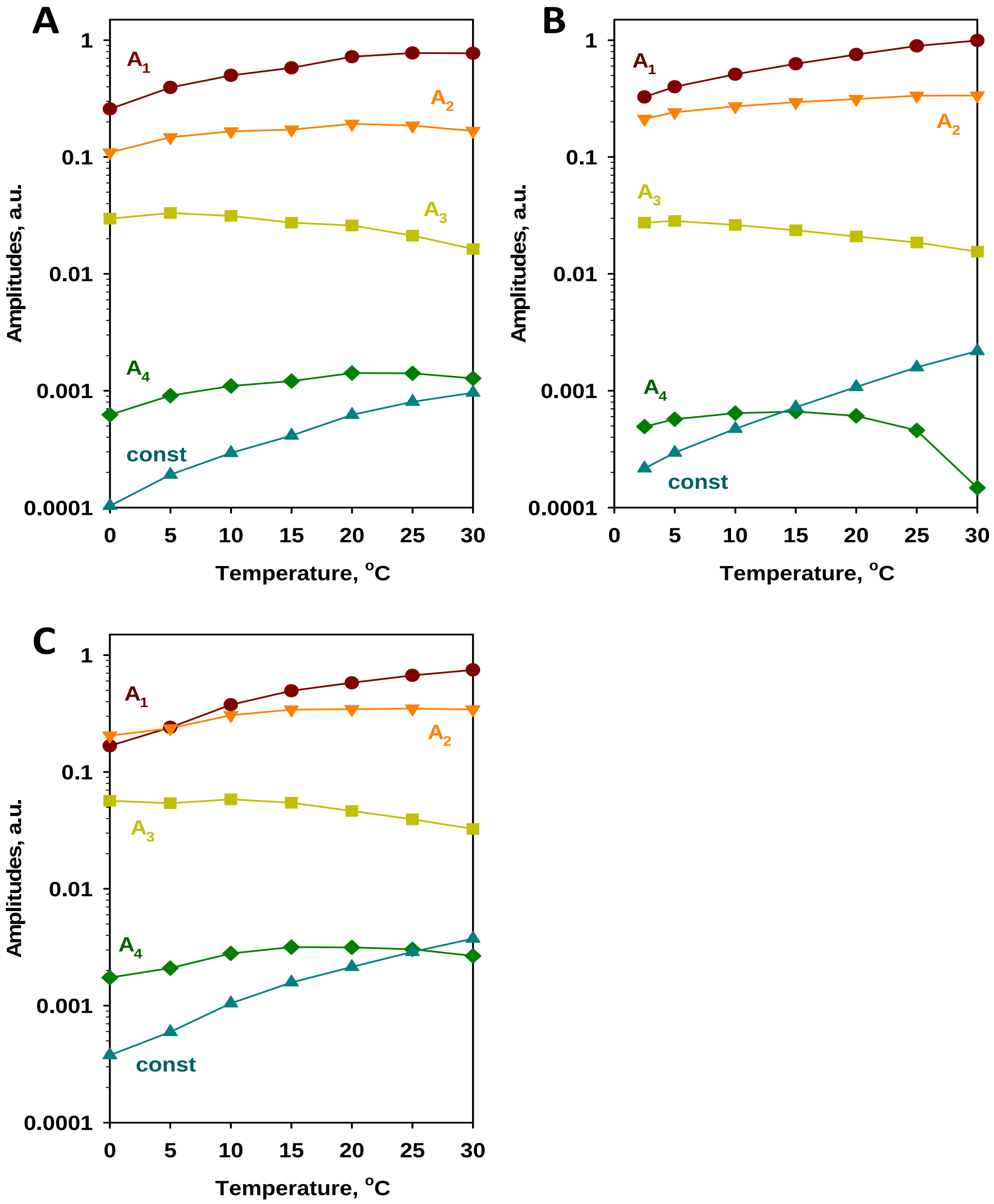

Supplementary Figure S8. Amplitudes of the *DF* decays measured after the second ns-Laser flash excitation of dark adapted PSII particles. (A) in H_2O buffer at pH 6.4. (B) in D_2O buffer at pD 6.4. (C) in H_2O buffer at pH 5.2. These simulation parameters are related to the second simulation, presented in Supplementary Figures S6, S7, S9 and Supplementary Table 1.

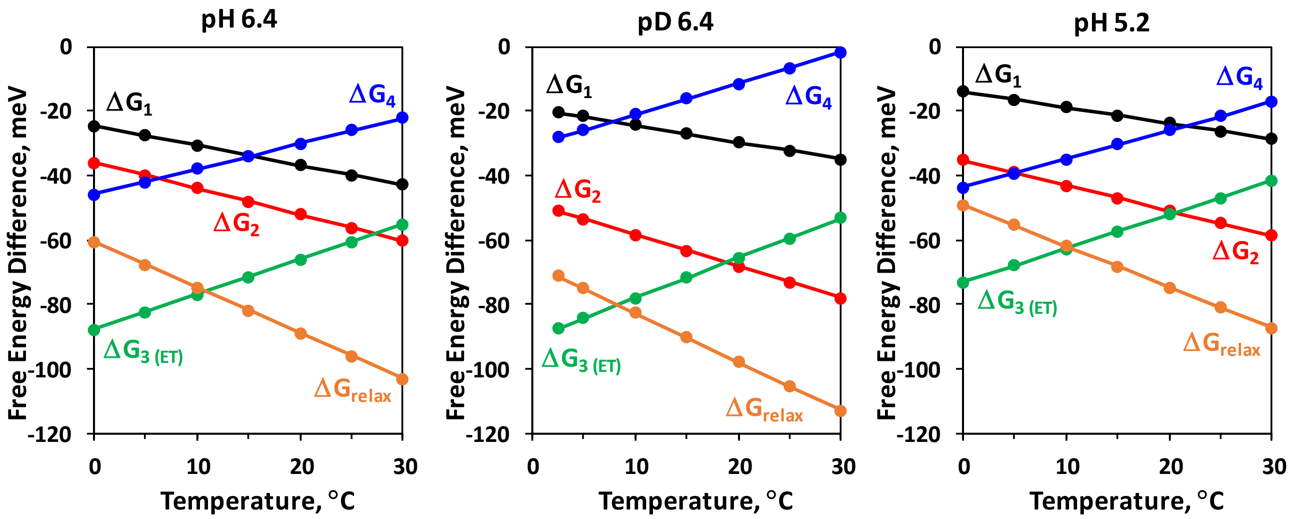

Supplementary Figure S9. Temperature dependence of the Gibbs energy, ΔG_i, of the individual kinetic components resolved in the *DF* decay after the second ns-Laser flash applied to dark adapted PSII particles. ΔG_{relax}, assignable to formation of a reaction intermediate after Y_Z oxidation but before the electron transfer from Mn_4CaO_5 complex is also shown (for calculation of ΔG_{relax} see Figure 2). These simulation parameters are related to the second simulation, presented in Supplementary Figures S6-S8 and Supplementary Table 1.

Supplementary Table 1. Time constants (τ), activation energies (*E_a*) and thermodynamic parameters Gibbs free energy (Δ*G*), Enthalpy (Δ*H*) and the entropic contribution to the change in Gibbs free energy (*T*Δ*S*) determined from simulations of the *DF* after the second ns-Laser flash applied to dark-adapted PSII membrane particles. Values for 20 °C are shown for the time constants, Δ*G*, and *T*Δ*S*. These simulation parameters are related to the second simulation, presented in Supplementary Figures S6-S9.

S-state transition	$S_2 \rightarrow S_3$	ET/PT	τ (μs)	E_a (meV)	ΔG (meV)	ΔH (meV)	-TΔS (meV)
H_2O, pH 6.4	$S_2^+ \rightarrow S_2^n$	H^+	18	0	-37	142	-179
			80	193	-52	185	-237
	$S_2^n \rightarrow S_3^+$	e^-	300	145	-66	-386	320
			1929	147	-30	-263	233
D_2O, pD 6.4	$S_2^+ \rightarrow S_2^n$	H^+	23	1	-30	125	-155
			103	236	-68	221	-290
	$S_2^n \rightarrow S_3^+$	e^-	480	203	-66	-431	366
			1861	232	-11	-294	282
H_2O, pH 5.2	$S_2^+ \rightarrow S_2^n$	H^+	23	0	-24	121	-145
			99	165	-51	179	-230
	$S_2^n \rightarrow S_3^+$	e^-	374	138	-52	-361	309
			1095	141	-26	-286	260

Supplementary Table 2. Simulation results for the delayed fluorescence decays recorded after the third saturating Laser flash excitation of dark-adapted PSII membrane particles. The confidence intervals were calculated from the covariance matrix of system, assuming that the degrees of freedom are equal to the chi2 of the system. We note that the calculated confidence intervals for the frequency factors (k_{oi}), and partially for the interrelated enthalpic (ΔH_i) and entropic (ΔS_i) changes are overestimated due to the specifics of the model (extrapolation to zero temperature where small deviations can result in big uncertainties in the parameter).

Parameter	Value	68.3% confidence interval	Value	68.3% confidence interval	Value	68.3% confidence interval
	Third flash, pH 6.4		Third flash, pD 6.4		Third flash, pH 5.2	
E_{a1}	46	48	47	44	158	26
E_{a2}	217	26	253	18	249	14
E_{a3}	204	49	258	55	294	44
E_{a4}	234	10	248	21	245	51
k_{01}	408	820	301	355	1.8×10^4	3.9×10^4
k_{02}	8.3×10^4	8.8×10^4	2.4×10^5	1.8×10^5	1.5×10^5	9.4×10^4
k_{03}	1.5×10^4	4.1×10^4	1.0×10^5	3.7×10^5	8.6×10^4	2.0×10^5
k_{04}	6.2×10^3	2.4×10^3	9.3×10^3	6.7×10^3	7.0×10^3	1.3×10^4
ΔS_1	-0.22	0.08	-0.25	0.07	0.08	0.05
ΔH_1	-82	24	-91	20	2	15
ΔS_2	0.70	0.24	0.85	0.33	0.33	0.05
ΔH_2	156	69	189	93	44	15
ΔS_3	-0.09	0.25	-0.23	0.33	-0.01	0.36
ΔH_3	-56	70	-107	93	-22	118
ΔS_4	0.39	0.07	0.35	0.07	0.11	0.30
ΔH_4	31	21	39	22	-32	96
a_4, 0 °C	0.00060	0.00006	0.00071	0.00007	0.00087	0.00019
a_4, 5 °C	0.00078	0.00006	0.00084	0.00007	0.00113	0.00021
a_4, 10 °C	0.00092	0.00005	0.00099	0.00006	0.00157	0.00023
a_4, 15 °C	0.00100	0.00004	0.00110	0.00005	0.00191	0.00022
a_4, 20 °C	0.00119	0.00003	0.00124	0.00004	0.00220	0.00019
a_4, 25 °C	0.00126	0.00003	0.00161	0.00004	0.00263	0.00017
a_4, 30 °C	0.00133	0.00003	0.00183	0.00005	0.00304	0.00016

Supplementary Table 3. Simulation results for the delayed fluorescence decays recorded after the second saturating Laser flash excitation of dark-adapted PSII membrane particles.

**Parameter**	**Value**	**68.3% confidence interval**	**Value**	**68.3% confidence interval**	**Value**	**68.3% confidence interval**
	**Second flash, pH 6.4**		**Second flash, pD 6.4**		**Second flash, pH 5.2**	
E_{a1}	49	14	47	24	23	42
E_{a2}	393	11	356	13	374	24
E_{a3}	355	12	417	18	393	24
E_{a4}	262	31	305	35	249	25
k_{01}	384	214	318	307	123	194
k_{02}	9.7×10^7	4.3×10^7	1.7×10^7	8.9×10^6	4.4×10^7	4.3×10^7
k_{03}	5.7×10^6	2.6×10^6	4.8×10^7	3.8×10^7	2.7×10^7	2.7×10^7
k_{04}	2.2×10^4	2.8×10^4	2.0×10^5	3.0×10^5	3.1×10^4	2.9×10^4
ΔS_1	0.14	0.06	0.03	0.07	-0.06	0.10
ΔH_1	10	18	-15	19	-32	29
ΔS_2	-0.20	0.10	0.13	0.10	-0.29	0.14
ΔH_2	-102	28	-20	27	-122	39
ΔS_3	-0.16	0.09	-0.16	0.32	-0.05	0.27
ΔH_3	-121	27	-103	98	-62	81
ΔS_4	-0.15	0.08	-0.62	0.25	-0.29	0.20
ΔH_4	-81	25	-219	77	-137	59
a_4, 0 °C	0.00011	0.00001	0.00024	0.00001	0.00040	0.00001
a_4, 5 °C	0.00020	0.00001	0.00032	0.00001	0.00062	0.00001
a_4, 10 °C	0.00031	0.00001	0.00050	0.00001	0.00110	0.00001
a_4, 15 °C	0.00043	0.00001	0.00075	0.00001	0.00166	0.00002
a_4, 20 °C	0.00064	0.00001	0.00110	0.00001	0.00223	0.00002
a_4, 25 °C	0.00082	0.00001	0.00161	0.00001	0.00300	0.00003
a_4, 30 °C	0.00097	0.00001	0.00221	0.00002	0.00388	0.00005

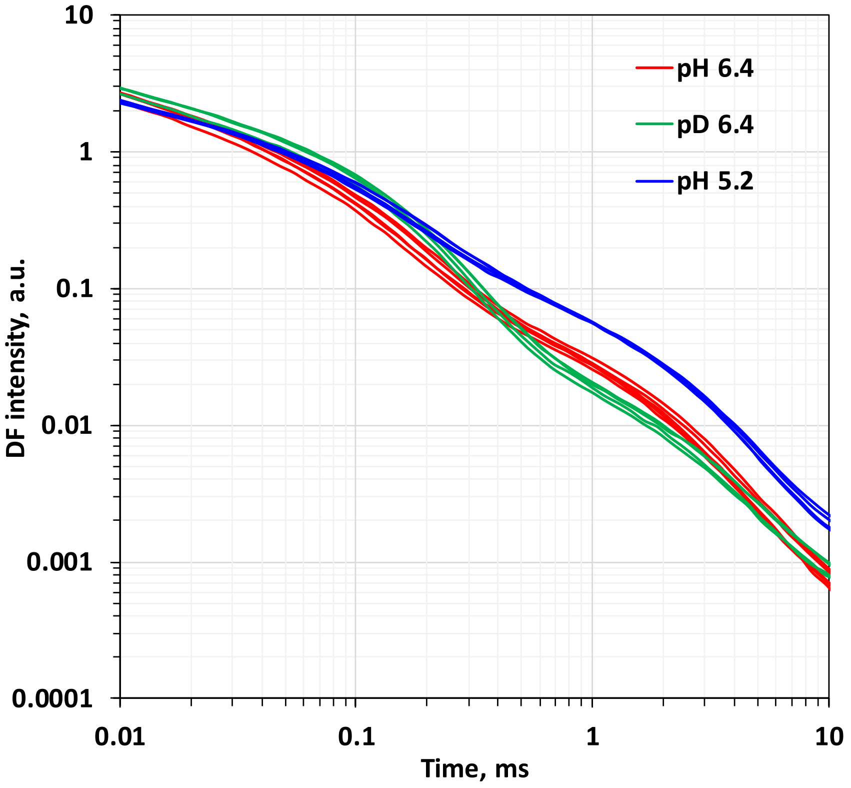

Supplementary Figure S10. Individual traces of DF decay recorded after the second ns-Laser flash excitation of dark adapted PSII samples in H_2O buffer with pH 6.4 (red lines), pH 5.2 (blue lines) and in D_2O buffer with pD 6.4 (green lines). The original data, not corrected for the artefact of the detector system and for the contribution from the [Q_A^-] decay are presented. The data were collected from several independent experiment, using different sample preps.
